# Screening for Viral Hemorrhagic Septicemia Virus in Marine Fish along the Norwegian Coastal Line

**DOI:** 10.1371/journal.pone.0108529

**Published:** 2014-09-23

**Authors:** Nina Sandlund, Britt Gjerset, Øivind Bergh, Ingebjørg Modahl, Niels Jørgen Olesen, Renate Johansen

**Affiliations:** 1 Research group Disease and Pathogen transmission, Institute of Marine Research, Bergen, Norway; 2 Research group Oceanography and climate, Institute of Marine Research, Bergen, Norway; 3 Section of Virology, National Veterinary Institute, Oslo, Norway; 4 Section of Virology, Technical University of Denmark, Frederiksberg C, Denmark; Kliniken der Stadt Köln gGmbH, Germany

## Abstract

Viral hemorrhagic septicemia virus (VHSV) infects a wide range of marine fish species. To study the occurrence of VHSV in wild marine fish populations in Norwegian coastal waters and fjord systems a total of 1927 fish from 39 different species were sampled through 5 research cruises conducted in 2009 to 2011. In total, VHSV was detected by rRT-PCR in twelve samples originating from Atlantic herring (*Clupea harengus*), haddock (*Melanogrammus aeglefinus*), whiting (*Merlangius merlangus*) and silvery pout (*Gadiculus argenteus*). All fish tested positive in gills while four herring and one silvery pout also tested positive in internal organs. Successful virus isolation in cell culture was only obtained from one pooled Atlantic herring sample which shows that today's PCR methodology have a much higher sensitivity than cell culture for detection of VHSV. Sequencing revealed that the positive samples belonged to VHSV genotype Ib and phylogenetic analysis shows that the isolate from Atlantic herring and silvery pout are closely related. All positive fish were sampled in the same area in the northern county of Finnmark. This is the first detection of VHSV in Atlantic herring this far north, and to our knowledge the first detection of VHSV in silvery pout. However, low prevalence of VHSV genotype Ib in Atlantic herring and other wild marine fish are well known in other parts of Europe. Earlier there have been a few reports of disease outbreaks in farmed rainbow trout with VHSV of genotype Ib, and our results show that there is a possibility of transfer of VHSV from wild to farmed fish along the Norwegian coast line. The impact of VHSV on wild fish is not well documented.

## Introduction

Viral hemorrhagic septicemia (VHS) is a severe virus infection causing great losses in farming of rainbow trout *Oncorhynchus mykiss*. Mortality rates are variable depending on the age of the fish with up to 100% in fry. It is often less in older fish, typically between 30–70% [1], [2]. Hence, economical losses could be substantial. VHS causes clinical signs such as haemorrhages in internal organs, pale gills, exophthalmia and darkening of the body [2].

The causative agent is the viral haemorrhagic septicaemia virus (VHSV), an enveloped negative single-stranded RNA virus belonging to the family of Rhabdoviridae and genus *Novirhabdovirus*. VHSV was first thought of as a virus affecting freshwater fish species of continental Europe. The first VHSV isolation from wild fish in the marine environment was in 1979 from Atlantic cod, *Gadhus morhua* [3], [4]. Since then the virus has proven to be both widely spread in the northern hemisphere and occurring in more than 80 marine and fresh water fish species [5]. This emphasizes the ability this virus has to adapt to new host species.

The four main genotypes of VHSV (I–IV) and the subtypes (a–e) are geographically distributed; genotype I, II and III are found in Europe while genotype IV occurs in North American and Northern Pacific waters [2], [6]. Subtype Ia is highly virulent for rainbow trout and this subtype is found in VHS infected fresh water farms in continental Europe. Subtype Ib has been isolated from several marine species in the Baltic and North Sea (reviewed in [1]) and has caused two outbreaks of VHS in rainbow trout in Sweden [7], [8]. Subtype Ic consists of older isolates from Denmark. Subtype Id represent another group of older isolates from the 1960s in Denmark and Norway as well as isolates from the more recent VHS outbreaks in rainbow trout farmed in sea cages in the Åland archipelago in the Baltic Sea [9]. Group Ie is only present in the Black Sea area. The Baltic Sea is also the main reservoir for the genotype II isolates. Genotype III has been isolated from wild marine fish in the North Sea and Skagerrak and has caused disease outbreaks in farmed turbot *Scophthalmus maximus* in Scotland and Ireland, in farmed rainbow trout in Norway [10] and recently also in wild caught farmed wrasse *Labridae* spp [11]. VHSV genotype IVa is widespread in wild marine fishes on both the American and the Asian side of the Northern pacific and has caused disease outbreaks in farmed Atlantic salmon in British Columbia, Canada [12]. It is still debated to what extent VHSV play a role in stock and population variations of Pacific herring *Clupea pallasii* ([13], reviewed in [14]). Over the past decade VHSV genotype IVb has become an emerging problem in the Great Lakes region in North America causing high mortality in several wild fish species [13], [15]–[18]. Subtype IVc has been isolated from brackish fishes during mortality events in Atlantic coastal regions of North America including mummichog *Fundulus heteroclitus*, stickleback Gasterosteidae, striped bass *Morone saxatilis* and brown trout *Salmo trutta* [12], [19].

Screening surveys of VHSV in wild marine fish have been performed at several separate locations in European waters; the Barents Sea, North Sea, Norwegian Sea, Skagerrak, Kattegat and Baltic Sea [20]–[24]. VHSV has been detected from the North Sea, coastal areas around Scotland, Skagerrak, Kattegat, the Baltic Sea and Flemish Cap, in the North Atlantic Ocean near Newfoundland and the virus is assumed endemic in these waters (reviewed in [1]). VHSV prevalence of up to 16.7% was found in Atlantic herring *Clupea harrengus* [21], [24], [25]. But most screening surveys performed on both Atlantic herring and Pacific herring [14], [26], [27] reports relatively few positive detections, in spite of high number of sampled fish (reviewed in [1]). Recently high prevalence of VHSV was reported in Atlantic herring from the southern part of Norway during the spawning season, however no disease symptoms associated with the findings were reported [28].

Although VHS outbreaks mainly occur in freshwater rainbow trout farms there have been a few reports of outbreaks of VHS in sea farmed rainbow trout in Sweden, Finland, France, Denmark and Norway [1], [7], [8], [10]. The VHS outbreak in Norway occurred in farmed rainbow trout in Storfjorden in 2007 [10]. This was the first detection of VHS in Norway since the eradication of the disease in 1974 and the source of the infection is still unknown. The outbreak in Norway was unexpectedly caused by a marine genotype III VHSV strain and challenge trials confirmed the marine virus-strain as virulent to rainbow trout [10], [29]. Before this VHSV genotype III was considered low pathogenic to rainbow trout and other salmonid fish species [30], [31]. This makes the outbreak unique and one of the main interests of the current screening was therefore to determine a possible marine reservoir in wild marine fish populations.

The Norwegian fish farming industry, especially the production of Atlantic salmon *Salmo salar*, has grown rapidly over the last decades. The annual production of Atlantic salmon and rainbow trout is now approx 1.2 mill and 70.000 tons respectively according to Statistics, Directorate of Fisheries in Norway (http://www.fiskeridir.no/). The possibility of pathogen transmission between farmed and wild fish is therefore increasing and a major concern. VHSV has so far not been isolated from Atlantic salmon in northern European waters, but VHSV genotype IVa has been isolated from farmed Atlantic salmon on the east coast of Canada (British Columbia) [15]. In general, Atlantic salmon has shown limited susceptibility to VHSV in immersion trails, but using intra peritoneal (i.p.) injection as challenge model has resulted in up to 78% mortality [1], [32]. In challenge experiments exposing Atlantic salmon to the genotype III VHSV isolate from Norway, mortality was only experienced in the i.p. injected fish group and not in the immersion trail groups [10]. However, recent work demonstrated that Atlantic salmon were susceptible to VHSV genotype IVa and developed clinical signs after i.p. injection, immersion and when cohabited with VHSV diseased Pacific herring [33]. Transmission of VHSV from infected Atlantic salmon to sympatric Pacific herring was also demonstrated. This shows that VHSV has the ability to adapt to and infect Atlantic salmon. It should be added that Atlantic salmon seems less susceptible to VHSV genotype IVb than IVa [34].

With more than 80 susceptible species in the marine and freshwater environment, VHSV shows high ability of host adaptation and evolution of new strains with increased virulence. VHSV is thereby a potential risk to farming of susceptible species. Evidence support the theory that both wild and farmed fish can function as a reservoir and transmitter of VHSV [11], [15], [33], [35], however more knowledge regarding specific species is needed. The presence of fish species that are asymptomatic carriers of VHSV also needs to be considered. Recently the Shetland Isles experienced an outbreak of VHS in wild caught wrasse held at a marine farm before use as cleaner fish in Atlantic salmon farming. The origin of this VHSV genotype III was likely from the marine environment, as several marine species in and around the locality tested positive for this virus [11]. As use of wrasse as cleaner fish is increasing, this points to another potential route of pathogen transmission to Atlantic salmon.

Screening of wild fish in northern parts of the Atlantic Ocean has earlier mainly been done in the open waters away from the aquaculture facilities. The aim of the present study was to investigate the occurrence of VHSV in marine wild fish in the coastal and fjord areas of Norway by analysing organ samples using both virus isolation and PCR.

## Material and Methods

### Field collection of fish

Five different research cruises in the fjords and coastal areas in Norway were carried out during 2009–2011. All cruises were part of the Institute of Marine Research annual coastal surveys and the fish samples used for VHSV analysis were randomly selected among the fish from the trawl haul. The Institute of Marine Research is a governmental research institute with given permission to perform research cruises including fish samplings by the Norwegian Government. Hence no additional permits were needed to sample organs from already deceased fish. All sampling and handling of fish were performed by experienced personnel.

**Figure 1:**
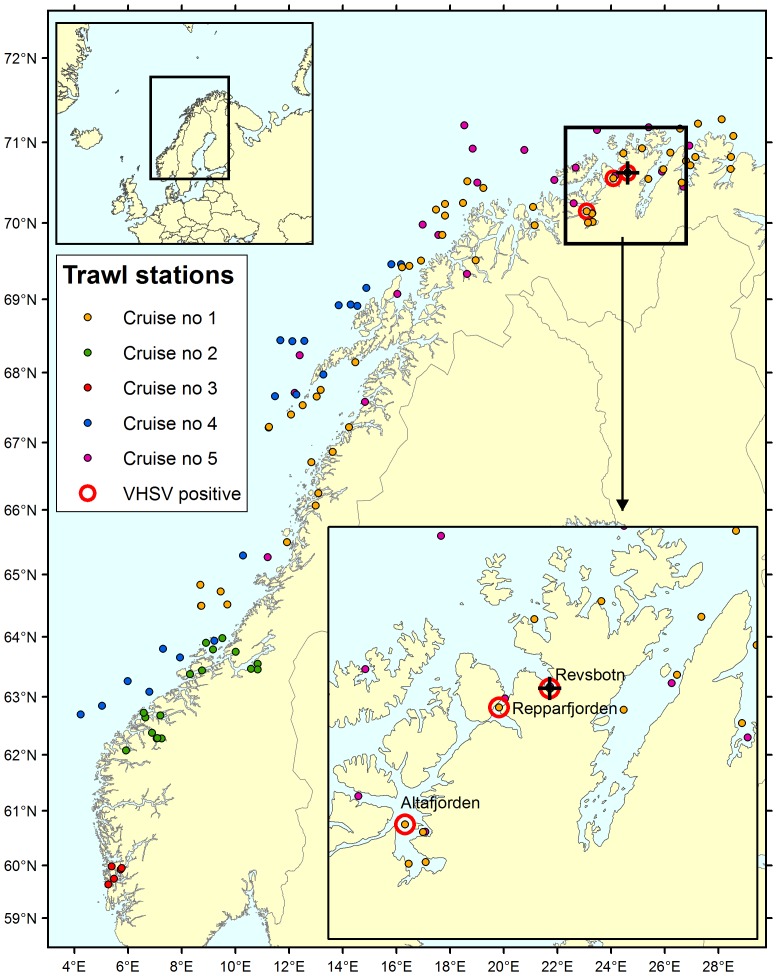
Map showing sampling locations (trawling stations) from cruises 1–5 indicated in colour. The various cruises are presented with different colour codes. Trawling stations with VHSV positive rRT-PCR samples are shown with red circles. The respective latitude/longitude for the locations are; Repparfjorden/Sammelsundet (70.5600/24.0800), Revsbotn (70.62667/24.6150) and Altafjorden (70.14667/23.0950). The black cross shows origin of the one pool sample testing positive by cell culture isolation.

Cruise one took place in September/October 2009 targeting the northern coast area. Cruise two was carried out in December 2009 targeting the coast of mid Norway including the Storfjorden fjord system were a VHSV outbreak occurred in farmed rainbow trout in 2007. Cruise three took place on the west coast in May 2010. During cruise four fish was sampled from the coast of north and mid Norway in October/November 2010. Cruise 5 took place in October/November 2011 in mostly the same areas as cruise one. The sampling area and geographical positions of each trawling station are shown in Figure 1.

During cruises one, two, four and five fishing was performed as either bottom or pelagic trawling, with trawling time of approximately 30 minutes. During cruise three only pelagic trawling was performed and it lasted up to 4 hours. Most fish died from suffocation in the trawling process or during handling on deck. All fish were kept in a cooled 4°C room until sampling and maximum time from fishing to sampling was 7–8 hours. During post mortem examination all fish were measured, weighed and any external signs of disease were recorded if present.

### Sampling of organs

All tissue samples were processed on board the research vessels. The same sampling procedure was used during all cruises. Organ specimens of spleen, kidney and brain from maximum 5 fish was pooled and diluted 1∶10 in transport medium (Eagle's Minimum Essential Medium, pH 7.6, supplemented with 10% newborn bovine serum and 100 µg ml^−1^ gentamicin). The samples were immediately transferred to a −80°C freezer for storage. Gonads (testes/ovaries) were sampled from sexually mature fish and kept in separate transport medium tubes. In addition individual samples of gills, heart, spleen, kidney, brain and gonads were collected in RNAlater (Sigma, USA), stored at 4°C for 24 hours prior to long term storage at −20°C. In cruise one the individual samples were randomly selected from three of the five pooled fish due to storage and sampling capacity. In cruise two-five individual samples were taken from all fish. Hearts were not individually sampled during cruise two, three and four due to storage capacity. Spleen and brain were diagonally sectioned and one half collected in transport medium and RNAlater for virus isolation and real-time Reverse Transcriptase Polymerase Chain Reaction (rRT-PCR) examination, respectively. The organs were aseptically sampled in this specific order; gills, heart, spleen, kidney and brain. If sexually matured gonads were present, these were sampled after the spleen. Equipment used to collect samples, were cleaned with alcohol, flamed between uses on each organ, and changed between each fish. Disposable gloves and tissue paper were changed between each fish. Equipment was washed and disinfected on daily basis to ensure sterile conditions; using Virkon S (Lilleborg, Norway) for minimum 30 minutes, rinsed in fresh water and boiled in fresh water for 15 minutes.

Following completion of the cruises, all samples were transported to the laboratory on dry ice and stored at either −20°C or −80°C prior to testing.

### Virus isolation

Tissue samples pooled in transport medium were homogenized and cleared by low-speed centrifugation, and supernatants inoculated onto subconfluent monolayers of BF-2-cells (ECACC, Salisbury, UK) in 1∶10 and 1∶100 dilutions in 24-well tissue culture plates according to the OIE procedure [5]. Inoculated cultures were incubated at 15°C and inspected after 1 week for cytopathic effect (CPE). Culture medium was collected from all wells and passed to new cell cultures. After a further week of incubation, the cultures were again inspected, and supernatant from wells with evident CPE in the second passage was collected, RNA extracted and tested for VHS-virus by rRT-PCR. Pooled organ samples that later were found VHSV positive by rRT-PCR were additionally incubated in BF-2 and EPC-cells three times for 14 days.

### RNA extraction and real-time RT-PCR (rRT-PCR)

RNA extraction was performed on homogenized tissue from organ pools (100 µl) and individual organ samples (10–20 mg), and from 150 µl virus supernatant using the automated easyMAG protocol (Biomérieux) or the RNeasy Mini kit (Qiagen). Extracted RNA was measured using NanoDrop ND-1000 (NanoDrop Technologies). The rRT-PCR assay was conducted using 500–1000 ng template RNA with a QIAGEN OneStep RT-PCR kit (QIAGEN Nordic) and nucleoprotein (N) gene primers and probe described by Duesund et al. [29]. The assay was performed with 0.5 µM of each primer and 0.3 µM probe in a 20 µl reaction, with cDNA synthesis at 52°C for 30 min followed by 15 min at 95°C, then 45 cycles of 95°C for 15 s and 60°C for 1 min using the Mx3005p real time PCR system (Stratagene). During the study the laboratory changed the standard VHSV rRT-PCR method to a validated assay with higher analytical and diagnostic sensitivity for all VHSV genotypes [36]. The assay was conducted using the same reaction conditions, but cycling conditions: 30 min a 50°C and 15 min at 95°C, followed by 45 cycles of 94°C for 30 s and 60°C for 1 min. Required positive and negative controls were included in all runs. Samples with specific cycle treshold (Ct) value ≤40 were considered positive.

### Sequencing and phylogenetic analysis

VHSV rRT-PCR positive samples were confirmed by sequence analysis of the viral G- and N-protein gene. Partial and full length glycoprotein (G) gene sequence was generated from overlapping sequences using primer sets V2, GB and Gseq [10], [37]. Three primers were used to obtain a 1217 bp nucleoprotein (N) gene sequence: N-G1F 5′-GCT CAC AGA CAT GGG CTT CA-3′, N-G2R 5′-TGG ATT GGG CTT CTT CTT-3′, N-G3F 5′-GGC TCA ACG GGA CAG GAA-3′. The RT-PCR was performed using 5 µl extracted RNA, 0.5 µM primer concentration in a 50 µl QIAGEN OneStep reaction, with cDNA synthesis at 50°C for 30 min followed by 15 min at 95°C, then 40 cycles of 95°C for 1 min, 55°C 1 min and 72°C for 90 s. The RT-PCR products were visualised on an agarose gel and purified using the ExoSAP-IT protocol (Usb) prior to sequencing with BigDye Terminator v3.1 Cycle Sequencing kit (Applied Biosystems). Sequences derived were aligned and compared to related VHSV sequences using Vector NTI Advance 11 (Invitrogen). A maximum-likelihood (ML) phylogenetic analysis was conducted using MEGA version 5.0 [38] on the complete G-gene alignment employing the GTR+G model. The obtained G- and N-gene sequences were deposited in GenBank and given accession numbers HM632035–HM632036 and KJ768664–KJ768665 for the isolate from Atlantic herring and silvery pout, respectively.

## Results

### Fish sampling

During the five cruises, a total of 1927 fish representing 39 different species were caught and sampled at 121 different haul stations. A total overview of the various fish species sampled during the separate cruises, the number of pooled samples and geographical distribution is given in Table 1 and Figure 1. No fish showed any visible signs of clinical disease during the post mortem examinations.

**Table 1:** Species and number of fish and pooled organ samples from all five research cruises.

Family	Cruise 1		Cruise 2		Cruice 3		Cruice 4		Cruice 5		All cruises	
Species	No.of sampled fish	No. of pools	No. of sampled fish	No. of pools	No. of sampled fish	No. of pools	No. of sampled fish	No. of pools	No. of sampled fish	No. of pools	Total no. of sampled fish	Total no. of pools
**Ammodytidae**												
Small sandeel *Ammodytes tobianus*	10	2									10	2
Lesser sandeel *Ammodytes marinus*	6	2									6	2
**Anarhichadidae**												
Wolf-fish *Anarhichas lupus*	22	5							4	1	26	6
**Argentinidae**												
Argentine *Argentina silus*	176	37					9	2	5	1	190	40
**Belonidae**												
Garfish *Belone belone*					27	7					27	7
**Carangidae**												
Horse mackerel *Trachurus trachurus*	1	1					3	1			4	2
**Clupeidae**												
Atlantic herring *Clupea harengus*	170	37*¤					7	2	45	11	222	50*¤
**Cyclopteridae**												
Lumpsucker *Cyclopterus lumpus*			1	1	17	6					18	7
**Gadidae**												
Cod *Gadus morhua*	64	14	14	4					32	6	110	24
Blue whiting *Micromesistius poutassou*	104	24					5	1	5	1	114	26
Four-bearded rockling *Rhinonemus cimbrius*	1	1									1	1
Haddock *Melanogrammus aeglefinus*	121	26					5	1	15	3	141	30
Ling *Molva molva*	5	3	1	1			3	1			9	5
Norway pout *Trisopterus esmarkii*	336	68							5	1	341	69
Pollack *Pollachius pollachius*			2	1			4	1			6	2
Poor cod *Trisopterus minutus*	12	4	15	3			1	1			28	8
Seith *Pollachius virens*	52	14	29	7			23	5	5	1	109	27
Silvery pout *Gadiculus argenteus*	60	12*									60	12*
Whiting *Merlangius merlangus*	54	12					4	1	13	3	71	16
**Lophiidae**												
Anglerfish *Lophius piscatorius*	10	6	4	2							14	8
**Lotidae**												
Tusk *Brosme brosme*	11	9	4	1			9	2			24	12
Blue ling *Molva dypterygia*			3	2							3	2
**Merluccidae**												
Hake *Merluccius merluccius*	2	1	23	6					4	1	29	8
**Osmeridae**												
Capline *Mallotus villosus*	79	16							10	2	89	18
**Phycidae**												
Greater forkbeard *Phycis blennoides*	3	1	3	1			4	1			10	3
**Pleuronectidae**												
Halibut *Hippoglossus hippoglossus*	1	1									1	1
Lemon sole *Microstomus kitt*	11	3									11	3
American plaice *Hippoglossoides platessoides*	10	2							5	1	15	3
Plaice *Pleuronectes platessa*	17	4	7	2			5	1	5	1	34	8
Witch *Glyptoceophalus cynoglossus*	10	4	5	1							15	5
**Scombridae**												
Macerel *Scomber scombrus*					5	1					5	1
**Sebastinae**												
Small redfish *Sebastes viviparus*	73	16	10	2			5	1			88	19
Golden redfish *Sebastes marinus*	20	5					5	1			25	6
*Sebastes* sp.	52	11									52	11
**Scophthalmidae**												
Megrim *Lepidorhombus whiffiagonis*	3	2	1	1							4	3
**Squalidae (dogfish sharks)**												
Piked dogfish *Squalus acanthias*			5	1							5	1
**Sternoptychidae**												
Pearlsides *Maurolicus muelleri*			2	1							2	1
**Triglidae**												
Grey gurnard *Eutrigla gurnardus*	4	2							3	1	7	3
**Zoarcidae**												
Eelpout *Lycodes vahlii gracilis*	1	1									1	1
Total number	1501	346	129	3	49	14	92	22	156	34	1927	453

Samples were found VHSV positive by virus isolation (¤) and rRT-PCR analysis (*).

The Atlantic herring caught during the research cruises is part of the Norwegian Spring Spawning herring (NSS) stock. The average length and weight of the herring caught at trawling station Repparfjorden was 15.7 cm (±2.9 cm STDV) and 40 g (±20.1 STDV) and at trawling station Revsbotn 22.7 cm (±3.4 cm STDV) and 124.5 g (±47 STDV). The individual length and weight of the positive fish, included in Table 2, do not differ significantly from the rest of the fish in the same catch (data not shown). Based on analysis of length and age distribution of NSS it can be estimated that the VHSV positive Atlantic herring were 4 years or less [39].

**Table 2:** VHSV rRT-PCR Ct values of pooled and individual organ samples from positive fish.

				Ct values individual organ samples						Ct values pooled organ samples	
Trawling location	Species	Length cm	Weight gr	Gills	Heart	Kidney	Spleen	Brain	Gonads	Pool no.	
Repparfjorden	Herring	16.0	44	28.48	24.75	33.61	31.23	33.03	N.A	63	37.17
Repparfjorden	Herring	17	42	37.44	32.27	-	-	-	-♀	63	37.17
Revsbotn	Herring	21.0	87	37.75	-	-	-	-	N.A	66	-
Revsbotn	Herring	25.5	164	38.32	-	-	-	-	N.A	67	-
Revsbotn	Herring	26.0	171	38.76	-	-	-	-	-♂	69	-
Revsbotn	Herring	27.0	200	37.68	-	-	-	-	N.A	70	33.17
Revsbotn	Herring	19.5	69	28.06	25.49	29.54	29.83	34.29	N.A	71	36.08
Revsbotn	Herring	17.0	44	28.21	28.96	34.06	31.8	36.53	N.A	72*	**26.74**
Revsbotn	Haddock	56.0	1152	**36.31**	-	-	-	-	N.A	73	-
Revsbotn	Haddock	44.5	849	**33.90**	-	-	-	-	N.A	74	-
Revsbotn	Whiting	40.0	586	**32.09**	-	-	-	-	-♀	75	-
Altafjorden	Silvery pout	17.0	47	37.62	**23.41**	37.9	39.99	38.17	N. A	102	37.65

VHSV rRT-PCR Ct values of separate organ samples from positive individuals, in addition to the corresponding pooled organ sample in which the individual samples were included. Fish length, weight and trawling location, from which each individual were caught, are included. Pool no. = identity pool number Herring = Atlantic herring *Clupea harengus*, silvery pout *Gaduculus argenteus*, haddock *Melanogrammus aeglefinus*, whiting *Merlangius merlangus*. - = No Ct. N.A = not available.

* = sample positive for VHSV by both virus isolation and rRT-PCR. VHSV sequences for verification were obtained from the samples indicated in bold.

### Cell culture isolation

One VHS-virus isolate were obtained from the 453 organ pools tested using BF-2 cell culture inoculation (Tables 1, 2). The positive pool contained organs from five Atlantic herring *Clupea harengus* collected during cruise one at location Revsbotn in Finnmark county (Figure 1). Full CPE was observed within 2 weeks of incubation, and VHSV was confirmed in the culture supernatant by rRT-PCR (Tables 1, 2). No CPE was observed in the other virus cultures. Organ pools later found VHSV positive by rRT-PCR were additionally sub-cultivated with prolonged incubation time without any detection of CPE.

### VHSV rRT-PCR detection

rRT-PCR screening of the 453 pooled samples revealed totally five VHSV positive pools (Table 2), four originating from Atlantic herring and one from silvery pout *Gadiculus argenteus*, all sampled on three trawling locations in Finnmark county within two days (Figure 1). Each pool consisted of organs from five fish. To follow up these positive findings all available organ samples from the Atlantic herrings and silvery pout represented in the pools (gills, heart, kidney, spleen and brain) were tested individually by rRT-PCR (Table 2). This revealed 1–2 positive fish per pool; respectively five Atlantic herring and one silvery pout.

Generally the Ct values obtained from the individual organs samples in positive fish show that the highest amount of VHSV RNA is present in the heart while gills showed the highest prevalence (Table 2). All individually sampled gills (n = 1369) and hearts (n = 1091) from all cruises were therefore tested by rRT-PCR. Due to low RNA yields on some of the gill samples (n = 183) results are only recorded from 1186 gills to avoid false-negative results (Table 2). Three additional Atlantic herring, two haddock *Melanogrammus aeglefinus* and one whiting *Merlangius merlangus* tested positive in gills, and they were all caught in the same trawl as the positive herring. The remaining sampled organs from these individual tested negative.

### Sequencing

**Figure 2:**
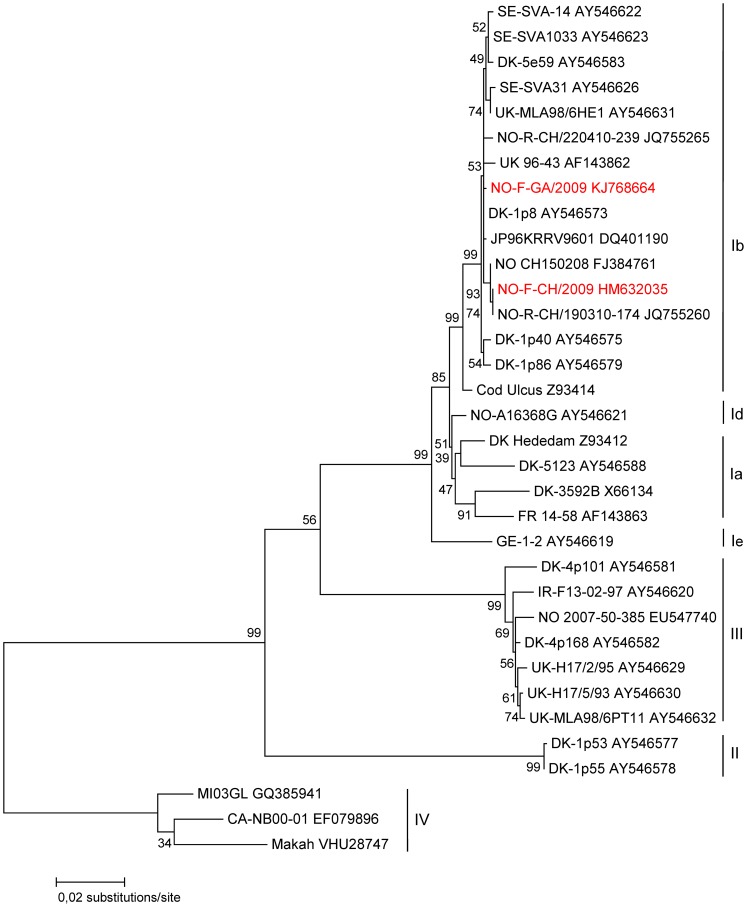
Maximum Likelihood (ML) phylogenetic tree showing the relationship of the new VHSV G-gene sequences (indicated in red) and other VHSV genotype representatives. Sequences are labelled by isolate name and GenBank accession number. The obtained tree was bootstrapped with 500 replicates and genotype IV was used as outgroup.

Unique G and N gene sequences were obtained from the PCR positive whiting, haddock, herring and silvery pout marked in bold in Table 2. This confirmed that they all belonged to genotype Ib and were closely related (99–100% identity, 6 nucleotide difference in the complete G-gene region). The partial sequences from whiting and haddock were too short to be included in the phylogenetic analysis. A ML phylogenetic tree based on complete G-gene sequences group the silvery pout and herring from this study together with other genotype Ib isolates reported from Atlantic herring and other fish species in the North Sea (Figure 2). The genotype Ib group also includes G-gene sequences detected in Atlantic herring of the Norwegian spring-spawning stock (Acc. no. JQ755260, JQ755265) [28].

## Discussion

Sampling during five research cruises was conducted to investigate the presence of VHSV in wild fish along the Norwegian coastline. The present screening survey is the first to look for VHSV in coastal areas and fjord systems in Norway. In total, samples from 12 fish including Atlantic herring *Clupea harengus*, silvery pout *Gadiculus argenteus*, haddock *Melanogrammus aeglefinus* and whiting *Merlangius merlangus* were found positive for VHSV. The VHSV positive fish were sampled on geographically close trawling locations in the northern county of Finnmark. This is the first observation of VHSV this far north, which again points out the large natural marine reservoir for VHSV.

To our knowledge this is the first report of sampling and testing for VHSV in silvery pout. Silvery pout is a small deep water fish of maximum 15 cm length that lives in the Northeast Atlantic and is used for industrial purposes such as fish meal and fish oil. Both Atlantic herring and silvery pout occur in large shoals which can migrate long distances. It is therefore uncertain whether the VHSV-infected fish were infected in Finnmark or elsewhere, and then migrated into the sampling area. It is interesting to note that these two highly different fish species are found in the same area carrying closely related virus isolates (Figures 1, 2).

The haddock and whiting only tested positive for VHSV in gills and it has not been confirmed if these fish where truly infected or only passive carriers of the virus in the gill mucus. None of the fish testing positive for VHSV in this study showed any clinical signs of disease. This is in line with numerous other isolations of VHSV from asymptomatic wild marine fish; including Atlantic herring caught in the English Channel [40], North Sea [21], Baltic Sea [24], [25] and Skagerrak and Kattegat [24]. The first marine isolate of VHSV was isolated in Danish coastal waters in 1979 from cod showing “ulcus syndrome”, however no evidence of an association between ulcers and VHS have been demonstrated [3], [4], [21], [24], [25], [41].

During the five research cruises samples were taken from 39 different species. The number of fish of each species is highly variable due to the fact that both bottom and pelagic trawling was used, and the trawling was performed in various areas at different times of the year. In addition, fish were sampled at random from random hauls and the need for fresh samples had to be prioritised. As high prevalence of VHSV has been found in Atlantic herring on previous screenings, Atlantic herring were prioritised for sampling when available [21], [24], [25], [28].

The 453 pooled organ samples (spleen, kidney and brain) were tested both with cell culturing and rRT-PCR, revealing a higher detection rate with rRT-PCR. Successful virus isolation in cell culture was only obtained from one pool of organs, including samples from five Atlantic herring. This sample was the strongest positive when tested using rRT-PCR (Ct 26), reflecting that overall the viral amount is relatively low. In addition, individual organs (spleen, kidney, brain, heart, gills and gonads) were sampled from most fish. rRT-PCR testing indicates that the highest amount of VHSV RNA was found in the heart and the highest prevalence were detected in gills. Heart samples are suitable for detecting VHSV in various fish species as it is an important target organ for VHSV [18], [42]–[45]. According to OIE (Commissions decision 2001/183) heart and/or brain samples should be included in screenings surveys. Based on our results from this present screening survey and previous findings, it is suggested that heart samples should always be included when sampling marine fish species.

Seven fish, including Atlantic herring, haddock and whiting, tested positive for VHSV by rRT-PCR in gills only. Gills have in various species proven to be a good organ for detecting VHSV carrying fish [28], [43], [46]. The results obtained by Cornwell et al. [46] further indicate that the sensitivity of detecting VHSV in gill samples might vary between species. The ability of VHSV to infect the gills has also been correlated to virulence [47]. A study testing the viral load of VHSV in various tissues of rainbow trout at various stages during the course of infection showed that fish surviving a VHSV infection had the highest amount of virus in gill and brain tissue [48].

This study detected a low prevalence of VHSV in Atlantic herring (8 positive/222 sampled) and this is in accordance with several other surveys in the waters in the northern part of Europe (reviewed in [1], [21], [24], [25]). High prevalence in Atlantic herring on the west coast of Norway were found during the spawning season in 2010 [28]. Whether this was caused by a generally higher prevalence during spawning seasons or an outbreak in this population is unknown and needs to be further investigated. Isolation of VHSV from Pacific herring *Clupea pallasii* populations is well known [14], [26], [27]. Studies have come to contradictory conclusions whether VHSV play a role in stock variations of Pacific herring or not ([13], reviwed in [14]).

There are indications that age plays an essential role in the susceptibility of VHSV. Higher prevalence of VHSV has been found in young wild caught Pacific herring compared to older [14], [49]. Another explanation can be that herring are infected during the early life stages and that the virus amount decrease over time to a non-detectable level in surviving fish. In captivity asymptomatic Pacific herring larvae and juveniles has developed VHS within a week after confinement [26] indicating a latent virus infection in the fish triggered by and developed in captivity. These findings supports the suggestion that young herring is more susceptible to VHSV infection than older. The age of the Atlantic herring tested in this study was estimated based on length and age distribution of NSSH [39]. This indicates that the VHSV positive Atlantic herring were less than 4 years. It can also be noted that the positive Atlantic herring samples were among the smallest specimen tested from each catch, and these results may be consistent with young herring being more susceptible to VHSV. In the present study the low amount of virus found and lack of clinical signs on the VHSV positive fish indicates that these individuals are asymptomatic carriers of the virus.

Few detections of VHSV from previous screening surveys could be related to methodology. According to Dixon [50] some VHSV isolates prefer BF-2 cells, while others produce highest titers in EPC cells. Isolation of marine VHSV, both of genotype Ib and III, is more successful in BF-2 cells compared to EPC [5], which was therefore the choice of cells used in the present study. Earlier screening surveys in wild marine fish have mostly tested pooled organ samples by cell culture isolation [21], [24], [25]. One exception is the screening performed by Dixon et al. [23] in which cell culture supernatant or dilutions of homogenized tissue from pooled organ samples were tested by RT-PCR. As in the present study an increased number of positive pooled samples (n = 4) were found using RT-PCR compared to cell culture isolation. Experimental testing of the sensitivity of cell culturing versus PCR-methodology has demonstrated RT-PCR the most sensitive [51], [52]. The development of PCR assays with higher sensitivity and a broader detection range to several genotypes of VHSV has made this the preferred method for VHSV detection in most laboratories [36].

Screening for VHSV based on testing of individual organs from marine fish is not common. Pooling of organ samples lower the sensitivity of the detection methods but at the same time allows testing of a larger amount of samples. Testing of all individual organs sampled during this study (n = 6848) was not feasible in our laboratory. The decreased sensitivity of the pooling strategy has been partly compensated by individual testing of all gill and heart samples. All individual organs from all available fish in the VHSV positive pools where tested by rRT-PCR revealing 1–2 positive fish per pool. This illustrates the difficulty of estimating prevalence based on pooled organ samples when virus yield and prevalence is low. The results further indicate that individual organ samples tested by rRT-PCR provide the best estimate for a true prevalence in the population. This difference in methodology may have led to an underestimation of the prevalence of VHSV in the European marine environment as methods with high sensitivity is required to detect carrier fish with low viral titers. Due to sampling and storage capacity during cruise one only three out of the five individuals included in each pool were sampled on RNAlater (n = 943). This could have affected the total number of positive results as additional positive individuals could have been included in the pool.

Sequencing based on the G and N gene revealed that the positive samples belonged to VHSV genotype Ib and were closely related. The ML phylogenetic tree group the strain from silvery pout and herring with isolates occurring in various fish species in the North Sea and Baltic Sea, including strains detected in Atlantic herring of the Norwegian spring-spawning stock [12], [28]. No genotype III positive fish were found in the present sampling, but such positive haddock and whiting are known from previous screenings in the North Sea [37]. The positive fish originated from sampling at three close trawling locations. The transmission pattern of VHSV from fish to fish is by direct contact, in water or by ingestions of infected material [53], [54]. In theory virus could transmit between fish while in the trawl. This is unlikely since internal organs also tested positive, indicating true carrier status. In addition, with one exception all individuals testing positive for VHSV came from different pool of fish, showing that the possibility of contamination between samples is limited. All positive pools also contain several negative fish showing that contamination was limited.

The risk of inter-species transmission of VHSV is always present in the marine environment. Evidence of VHSV transmission from wild to farmed fish has recently been studied by phylogenetic analysis that verified a closely genetic linkage between VHSV genotype IVa isolates from wild and farmed fish in the British Columbia area [15]. In the fjord systems were the positive marine samples were found in this study both wild and farmed Atlantic salmon are present (data from the Directorate of Fisheries). In addition Altafjorden and Repparfjorden are regulated fjord systems established for the protection of wild salmon. Although Atlantic salmon in general has shown low susceptibility to VHSV, relatively high virus titer (between 1×10^4^ and 1×10^6^ pfu/g) of VHSV was demonstrated in Atlantic salmon 10 weeks after i.p. injection, immersion and cohabitation challenge with VHSV genotype IV [33]. Three of 12 Atlantic salmon i.p. injected with the genotype III isolate from Storfjorden were still VHSV positive 29 days after infection [10]. This susceptibility and persistency of VHSV in Atlantic salmon demonstrated by Lovy et al. [33] together with the possibility of transmitting the virus back to Pacific herring indicates that salmon has the ability to serve as a vector and reservoir of VHSV. Although VHSV genotype III isolates does not normally cause disease in anadromous fish species, the outbreak in rainbow trout in Norway in 2007 shows the adaptation capacity of this virus for salmonids [10]. Therefore, it is important to keep farmed fish free of any VHSV by avoiding continuous production of the same fish species for several generations and keep generations at separate locations. The possibility of inter-species transmission of VHSV between fish species in close contact is highly relevant especially with the increased use of cleaner fish as biological control of sea lice in Atlantic salmon farms. Although all Atlantic salmon from a farm site that experienced an outbreak of VHSV on wrasse tested negative for VHSV, the potential of VHSV to adapt and cause disease outbreaks in Atlantic salmon farms, should be taken into account [55].

The fish testing positive for VHSV in the current study appeared asymptomatic carriers. The healthy carriers could represent survivors from an earlier disease outbreak with high mortality rates. Mortality rates in wild fish populations are often not detected due to removal of diseased fish by predators [56]. It is also possible that the virus carrier fish is weakened and thereby more susceptible to other disease problems or predators. Little is known about how VHSV affect the health situation of wild fish populations and further research is needed. Possible transfer of VHSV between wild and farmed fish will always be a potential risk and knowledge about marine reservoirs is therefore essential. It is therefore of major importance to conduct surveillance studies for VHSV to ensure early detection and eradication of the virus from farmed fish. History has taught us that such control is important to avoid adaptation of the virus into more virulent strains.
